# The effect of lifestyle on late-life cognitive change under different socioeconomic status

**DOI:** 10.1371/journal.pone.0197676

**Published:** 2018-06-13

**Authors:** Pei-Hsuan Weng, Jen-Hau Chen, Jeng-Min Chiou, Yu-Kang Tu, Ta-Fu Chen, Ming-Jang Chiu, Sung-Chun Tang, Shin-Joe Yeh, Yen-Ching Chen

**Affiliations:** 1 Department of Family Medicine, Taiwan Adventist Hospital, Taipei, Taiwan; 2 Institute of Epidemiology and Preventive Medicine, College of Public Health, National Taiwan University, Taipei, Taiwan; 3 Department of Geriatrics and Gerontology, National Taiwan University Hospital, Taipei, Taiwan; 4 Institute of Statistical Science, Academia Sinica, Taipei, Taiwan; 5 Department of Neurology, National Taiwan University Hospital, Taipei, Taiwan; 6 Department of Public Health, College of Public Health, National Taiwan University, Taipei, Taiwan; 7 Research Center for Genes, Environment and Human Health, College of Public Health, National Taiwan University, Taipei, Taiwan; University Of São Paulo, BRAZIL

## Abstract

This study aimed to identify lifestyle factors associated with cognitive change and to explore whether the effect of lifestyle varies by socioeconomic status (SES). Participants aged 65 years and older were recruited from elderly health checkup programs from 2011 to 2013 in Taiwan. Neuropsychological tests, including tests of global cognition, logical memory, executive function, verbal fluency and attention, were administered at baseline (N = 603) and 2 years later (N = 509). After literature review, 9 lifestyle factors and 3 SES indicators were chosen and their effects on cognitive change were evaluated using linear regression adjusting for age, sex, education, *APOE* ε4 status, and baseline cognitive score. Five lifestyle factors (high vegetable and fish intake, regular exercise, not smoking, and light to moderate alcohol consumption) and 3 SES indicators [annual household income (> 33,333 USD vs. less), occupational complexity (high vs. low mental demanding job), and years of education (> 12 years vs. less)] were found to be protective against cognitive decline (*P* < 0.1 in any cognitive domains, ß ranging from 0.06 to 0.38). After further adjusting for all the lifestyle and SES factors, fish intake, higher income and occupational complexity remained protective. Significant interactions were found between a healthful lifestyle (defined as having ≥ 3 healthful lifestyle factors) and income on changes of global cognition and verbal fluency (*P*_interaction_ = 0.02 and 0.04). The protective effect of a healthful lifestyle was observed only among participants with lower income in global cognition and logical memory [ß = 0.17, 95% confidence interval (CI) = 0.07–0.26; ß = 0.30, 95% CI = 0.14–0.46]. To the best of our knowledge, this study for the first time explored how the interactions of lifestyle and SES affect cognitive change. Our findings will aid in developing dementia prevention programs and reduce health inequalities.

## Introduction

Dementia is one of the most common debilitating disorders in the elderly, with an estimated lifetime risk of 1 in 5 for women and 1 in 6 for men over 65 years of age [1]. Estimates indicate that delaying the onset of dementia by only five years may reduce the number of cases by one-third [2]. Therefore, identifying modifiable risk factors for cognitive decline is critical for the development of dementia prevention programs. It is also important to identify the most vulnerable group and who may benefit most from health promotion intervention.

Currently, well-known modifiable risk factors for dementia include vascular risk factors, depression, unhealthful lifestyle, and low socioeconomic status (SES) [3]. Most health intervention programs focused on enhancing healthful lifestyle. Previous meta-analyses have shown that the choices of healthful lifestyles, which include an increased intake of vegetables and fish, regular exercise and not smoking, are associated with decreased risk of dementia [3–8]. Additionally, frequent social activity and cognition-enhancing activity [9, 10], light to moderate alcohol consumption [11], and coffee or tea intake [12] have been associated with decreased risk for dementia or cognitive decline, although the results are less consistent for these factors.

Disadvantaged SES is also associated with an increased incidence of dementia and cognitive impairment [13–18]. Many studies suggested that the effects of SES on cognition are partly related to different lifestyles, because socioeconomically disadvantaged individuals tend to have poorer dietary quality [19] and more risky health behaviors [20]. Low SES may also directly affects cognition through a lower cognitive reserve related to lower levels of education or occupational complexity [21]. Furthermore, some studies found that SES may act as an effect modifier between lifestyle and health. Blaxter et al. found that healthful lifestyles reduce more deaths among high-SES individuals [22]. However, the recent evidence by Birch et al [23]. and Pampel et al. [24] showed the opposite: non-smokers had better self-reported health and less morbidity compared with smokers, and the benefits of not smoking were more pronounced among low-SES population. These heterogeneous results may be attributable to different health outcomes and varied social contexts.

Despite the numerous findings implicating close relationships between lifestyle and SES, how these factors interact and their overall effect on cognitive decline are poorly understood. Whether the effect of lifestyle on cognition differs according to SES remains unknown. This knowledge may be important in developing targeted dementia prevention programs and reducing health disparities. Besides, most previous studies focused on the effect of a single lifestyle factor on cognition. However, healthful lifestyles tend to cluster together or act synergistically, and thus they should be considered together. In addition, dietary habits and lifestyle patterns may vary widely by ethnicity, but few studies have evaluated the effects of multiple lifestyle factors in Chinese.

This study aimed to explore three research questions. First, we want to identify late-life lifestyle factors that are protective against cognitive decline in community-dwelling Chinese elderly. Second, to examine whether lifestyle and SES both have independent effects on cognitive change after controlling for each other. Third, to explore whether the effect of lifestyle on cognitive change varies by SES.

## Materials and methods

### Participants

This prospective cohort study recruited 603 community-dwelling elderly 65 years of age and older from the elderly health checkup programs at National Taiwan University Hospital (NTUH) from 2011 to 2013. Exclusion criteria were diagnosed dementia, severe diseases that may prohibit study participation such as advanced cancer or decompensated organ failure, marked dependency in activities of daily living (Barthal index less than 60 points), and marked loss of vision, hearing, or communicative ability. The final analysis included 509 individuals who completed a follow-up assessment 2 years later (reasons for loss to follow-up: death = 3, institutionalization = 3, severe disease or disability = 11, lost contact = 12, busy = 19, refusal to participate = 46). The 2-year follow-up period was determined according to previous studies examining the effect of lifestyle interventions on cognitive change in elderly [25, 26]. Written informed consent was obtained from each participant. The study protocol was approved by the institutional review board at NTUH (201101039RB, 201112047RIB). All of the experiments were carried out in accordance with the guidelines of the World Medical Association Declaration of Helsinki. A detailed questionnaire was administered via a face-to-face interview and included data on demographics, lifestyle, dietary intake, SES and comorbidities. All of the medications were recorded by direct inspection and classified by a physician to assist the ascertainment of comorbidities [e.g. hypertension and diabetes mellitus (DM)]. Genomic DNA was extracted from buffy coat of blood using the QuickGene-Mini80 kit (Fujifilm, Tokyo, Japan).

### Cognitive function assessment

A battery of validated neuropsychological tests were used to assess cognitive function [27], administered by trained examiners following a standardized protocol at baseline and two years later. Global cognition was measured by the following 11 tests of 4 cognitive domains. The logical memory was assessed through the logical memory subtests in Taiwanese version of the Wechsler Memory Scale-Third Edition (WMS-III) [28]: the immediate free recall and theme recall tests and the delayed free recall and theme recall tests. Executive function was measured with the trail-making A and trail-making B tests. Verbal fluency was assessed by naming fruits, fish and vegetables within 60 seconds [29]. Attention was assessed with the digit span forward and backward components of the WMS-III [28].

### Lifestyle assessment

We assessed 9 lifestyle factors associated with cognitive change, which were selected according to previous literature [3–12]. A semi-quantitative 44-item food frequency questionnaire (FFQ), which has been validated in Taiwan [30], was used to assess the participants’ typical dietary intake in the previous year. The details of the FFQ were provided in our previous work [31]. The reported intake frequency and portion size of food or beverage were converted to daily servings. In this study, the examined food groups included vegetables, fruits, and fish, which were tertiled for analysis [highest tertile of daily servings (T3) vs. lower tertiles (T1+T2)]. Coffee or tea intake was defined as habitual intake of either coffee or tea. Leisure activities included physical activity (T3 of weekly energy expenditure for each sex), as measured with the Taiwanese version of the International Physical Activity Questionnaire (IPAQ) [32]; social activity (≥ once/week) [33]; and cognition-enhancing activity (≥ 3 times/week) [33]. Not smoking was defined as no current smoking habit according to previous literature [8]. Light to moderate alcohol consumption was defined as habitual alcohol drinking but consumption of less than 2 units of alcohol per day (1 unit equals to 8 g of pure alcohol) [11, 34].

### Socioeconomic status

SES was characterized by 3 indicators: income, occupation, and education, which were obtained from self-report questionnaires. We asked the individuals to report the annual household income from all sources before taxes that they can actually share or use, because many Chinese share their properties with their spouses or parents. The income was recorded in four categories: <10,000 US dollar (USD); 10,000–26,666 USD; 26,666–33,333 USD; and >33,333 USD (the gross national income per capita of Taiwan was 19864 USD in 2010). The income data was collected in categories because people tend to under-report or refuse to answer if they were asked about the actual amount of income. Furthermore, these categories were dichotomized into low and high income, i.e., less and more than 33,333 USD, respectively. Occupation was dichotomized into lower mental demanding job with low complexity (housewife, unskilled/semiskilled, skilled trade or craft, or clerical/office worker) and high complexity (managerial, professional or skilled workers) according to the job of longest duration [15]. Education level was classified into high school education or less versus more than high school education (≤ 12 vs. > 12 years) [35].

### Covariates

The height, weight, and blood pressure were measured using standardized procedures and equipment. Body mass index (BMI) was calculated as the weight in kilograms divided by the square of the height in meters (kg/m^2^). The fasting glucose was included in the elderly health checkup program. Apolipoprotein E (*APOE*) ε4 was genotyped with TaqMan Genomic Assays using an ABI 7900HT fast real-time PCR system (Applied Biosystems Inc., CA, USA) and was determined by using the assay developed by Chapman *et al*.[36].

Regarding comorbidities, hypertension was defined as blood pressure ≥ 140/90 mmHg, self-reported hypertension, or use of antihypertensives. DM was defined as fasting glucose ≥ 126 mg/dL, self-reported DM, or use of hypoglycemic agents. Stroke was based on self-reported medical history. Depressive symptoms were assessed with the Chinese-version 20-item Center for Epidemiologic Studies Depression Scale (CES-D) [37].

### Statistical analyses

For each cognitive test, a Z score was computed for each participant at baseline and at follow-up, on the basis of the means and standard deviations (SDs) of the population test scores at baseline [25]. The use of Z scores allows comparisons between cognitive tests with different scales and examines changes over time, and was widely used in previous studies [25, 26]. In order to make all of the cognitive tests in the same direction with the degree of cognitive performance, the scores (seconds of completion the test) of the trail-making A and B tests were multiplied by -1 to obtain negative values such that higher scores represented better cognition. The Z scores of the cognitive tests within different cognitive domains were averaged to form domain-specific scores [38].

The primary outcome was domain-specific cognitive change, computed as the difference between the follow-up and baseline domain Z scores. We assessed the associations of each of the 9 lifestyle factors and 3 SES indicators with specific cognitive domain adjusting for age, sex, number of years of education, *APOE* ε4 status, and baseline domain score. If *P* < 0.10 was observed, the specific lifestyle factor or SES indicator was selected for further analysis. To test the linear trend between baseline characteristics and the number of healthful lifestyle factors, a Cochrane-Armitage test was performed for categorical variables [39], and general linear models were utilized for continuous variables. Comparisons of baseline characteristics between low and high SES individuals were also performed. A multivariable linear regression analysis was further used to examine the effect of the number of healthful lifestyle factors on cognitive changes after adjustment for age, sex, number of years of education, *APOE* ε4 status, baseline cognitive domain score, CES-D score, hypertension, DM, stroke, occupation, annual income, and daily energy intake (log-transformed due to a left skew). To examine whether the selected lifestyle factors and SES indicators have independent effects on cognitive change, multivariable linear regression models were used adjusting for the above covariates and each of the lifestyle factors and SES indicators.

Another goal of this study was to examine whether the effect of a healthful lifestyle on cognitive change varies according to SES. The statistical interactions between effects of having a healthful lifestyle (defined as having ≥ 3 out of 5 healthful lifestyle factors) and different SES indicators on cognitive change were examined after adjusting for the above covariates. All statistical analyses were performed using SAS 9.4 (SAS Institute, Cary, NC, USA). A two-sided *P* < 0.05 was considered statistically significant.

## Results

A total of 603 participants were recruited, and the final analysis included 509 individuals who completed follow-up after 2 years. The mean age at baseline was 73 years old, and 52.7% were females. The Z scores of global cognition, logical memory, and verbal fluency significantly decreased after 2 years (mean change in SD of the baseline population domain scores: -0.12, -0.20, -0.17, respectively; all *P* < 0.001). The executive function and attention did not change significantly (mean change: -0.04, 0.05). The descriptive statistics of lifestyle factors were shown in Table 1. The mean daily energy intake was 1584 ± 383 kcal/day. Most people in this population had relatively higher SES compared with the average of elderly in Taiwan. The mean years of education was 13.6 years, and 60% had more than 12 years of education. About 52% of participants had occupation with high complexity, and 85% reported they were retired. About 43% of participants had annual household income more than 33,333 USD.

**Table 1:** Descriptive statistics of the lifestyle factors and their cut-off points.

Lifestyle factors		Mean ± SD/N(%)	Cut-off point
Diet	Vegetables (servings/day)	2.70 ± 1.23	≥ 2.9 (T3)
	Fruits (servings/day)	3.18 ± 1.21	≥ 4.5 (T3)
	Fish (servings/day)	0.54 ± 0.35	≥ 0.8 (T3)
	Coffee or tea use (N, %)	293 (58%)	NA
Leisure activity	Physical activity (MET-minutes per week)		
	Men	2013 ± 1775	≥ 2118 (T3)
	Women	1505 ± 1299	≥ 1800 (T3)
	Social activity (frequency per week)	1.73 ± 2.13	≥ 1
	Cognitive activity (frequency per week)	6.83 ± 0.97	≥ 3
Smoking & alcohol	No current smoking (N, %)	494 (97%)	NA
	Light to moderate alcohol (N, %)	33 (6%)	> 0 to < 2 units of alcohol/day

The continuous lifestyle factors were dichotomized according to the above cut-off points. SD = standard deviation; T3 = tertile 3; NA = not applicable; MET, metabolic equivalent.

Five out of nine lifestyle factors with *P* < 0.10 in the multivariate model were selected for further analysis, which included high vegetable intake (T3: ≥ 2.9 servings/day), high fish intake (T3: ≥ 0.8 servings/day), high physical activity [T3: ≥ 2,118 metabolic equivalent (MET)-minutes per week for men; ≥ 1,800 MET-minutes per week for women], no current smoking (3%), and light to moderate alcohol consumption (6%) (Table 2). All three SES indicators were kept for further analysis because each of them showed significant association with different cognitive variables (Table 2).

**Table 2:** Effects of different lifestyle factors and SES indicators on cognitive change.

Lifestyle factors N (%)		Cognitive variables				
		Global cognition	Logical memory	Executive function	Verbal fluency	Attention
		ß (95% CI)	ß (95% CI)	ß (95% CI)	ß (95% CI)	ß (95% CI)
Diet	Vegetables^b^ 152 (30%)	0.04 (-0.02, 0.10)	0.18 (0.07, 0.30)**	-0.12 (-0.23, -0.02)*	0.02 (-0.08, 0.12)	0.01 (-0.08, 0.11)
	Fruits 169 (33%)	-0.01 (-0.07, 0.05)	0.003 (-0.11, 0.11)	-0.07 (-0.17, 0.03)	-0.02 (-0.11, 0.08)	0.02 (-0.08, 0.11)
	Fish^b^ 143 (28%)	0.06 (-0.0003, 0.13)^†^	0.09 (-0.03, 0.21)	0.06 (-0.05, 0.17)	0.02 (-0.08, 0.12)	0.07 (-0.02, 0.17)
	Coffee or tea 293 (58%)	-0.005 (-0.06, 0.05)	0.02 (-0.09, 0.12)	0.03 (-0.06, 0.13)	0.04 (-0.05, 0.13)	-0.03 (-0.12, 0.06)
Leisure activity	Physical act.^b^ 169 (33%)	0.03 (-0.03, 0.09)	0.10 (-0.01, 0.21)^†^	0.02 (-0.09, 0.12)	0.05 (-0.05, 0.14)	0.01 (-0.08, 0.10)
	Social act. 253 (50%)	-0.03 (-0.09, 0.03)	-0.06 (-0.17, 0.04)	0.02 (-0.08, 0.11)	-0.02 (-0.11, 0.07)	0.01 (-0.08, 0.10)
	Cognitive act. 494 (97%)	-0.10 (-0.27, 0.06)	-0.21 (-0.52, 0.09)	0.03 (-0.26, 0.32)	-0.07 (-0.33, 0.19)	-0.10 (-0.35, 0.16)
Smoking & alcohol	Not smoking^b^ 494 (97%)	0.16 (-0.002, 0.33)^†^	0.29 (-0.02, 0.60)^†^	0.13 (-0.16, 0.42)	-0.09 (-0.36, 0.17)	0.18 (-0.07, 0.44)
	Light to moderate alcohol^b^ 33 (6%)	0.10 (-0.01, 0.22)^†^	0.19 (-0.02, 0.41)^†^	0.18 (-0.02, 0.38)^†^	0.12 (-0.07, 0.30)	-0.03 (-0.21, 0.15)
Socio- economic status	Higher income^b^ 208(43%)	0.15 (0.10, 0.21)***	0.38 (0.28, 0.49)***	-0.04 (-0.15,0.06)	0.03 (-0.06, 0.13)	0.02 (-0.07,0.11)
	Higher occupational complexity^b^ 264 (52%)	0.04 (-0.02, 0.10)	0.18 (0.06, 0.29)**	-0.08 (-0.19, 0.02)	-0.02 (-0.11, 0.08)	0.05 (-0.04, 0.15)
	Higher education^b^ 304 (60%)^a^	0.03 (-0.04, 0.09)	0.15 (0.03, 0.27)*	0.02 (-0.08, 0.13)	0.03 (-0.06, 0.13)	0.13 (0.03, 0.23)*

The effects of individual healthful lifestyle factors on cognitive change in different domains are presented after adjustment for age, sex, number of years of education, *APOE ε*4 status, and baseline cognitive domain score.

^a^Adjusted for age, sex, *APOE ε*4 status, and baseline cognitive domain score.

^b^ Lifestyle factors with *P* < 0.10, which were selected for further analyses.

Higher income: annual household income > 33,333 USD. Higher occupational complexity: occupation with higher mental demands. Higher education: education > 12 years. CI = confidence interval; act. = activity.

^†^*P* < 0.1,

*P < 0.05,

**P < 0.01,

***P < 0.001.

Comparing the baseline characteristics, participants with a higher number of healthful lifestyle factors had fewer depressive symptoms, had better global cognition and attention at baseline, and had higher household income (*P*_*trend*_ < 0.05, Table 3). Participants with a lower income had more depressive symptoms and less vegetable intake compared with economically advantaged individuals (S1 Table). Those with lower occupational complexity and a lower education level were more likely to be female. The distribution of lifestyle factors were not different between elders with different occupational complexity or education levels (S1 Table). A graded relationship was observed between more numbers of healthful lifestyle factors and less decline in global cognition and logical memory after adjusting for covariates (*P*_*trend*_ = 0.02 and 0.004, respectively, S1 Fig).

**Table 3:** Baseline population characteristics by number of healthful lifestyle factors.

	Number of healthful lifestyle factors			
	0–1 (N = 183)	2 (N = 189)	3–5 (N = 132)	*P*_*trend*_
	**Mean ± SD**			
Age (years)	73.9 ± 5.7	72.3 ± 5.1	72.9 ± 5.4	0.06
Education (years)	13.6 ± 3.8	13.6 ± 3.8	13.7 ± 3.7	0.72
BMI (kg/m^2^)	24.1 ± 3.3	23.8 ± 3.0	24.0 ± 2.7	0.73
CES-D score	3.4 ± 6.6	2.9 ± 6.0	1.9 ± 4.7	0.04
Global cognition	-0.08 ± 0.69	0.04 ± 0.62	0.10 ± 0.61	0.01
Logical memory	-0.06 ± 0.94	0.004 ± 0.94	0.10 ± 0.89	0.13
Executive function	-0.10 ± 0.94	0.07 ± 0.84	0.06 ± 0.79	0.10
Verbal fluency	-0.10 ± 0.90	0.06 ± 0.80	0.06 ± 0.86	0.08
Attention	-0.08 ± 0.88	-0.01 ± 0.84	0.15 ± 0.81	0.02
	**N (%)**			
Female sex	93 (51%)	112 (59%)	60 (45%)	0.48
*APOE* ε4 carriers	31 (17%)	28 (15%)	25 (19%)	0.69
Hypertension	133 (74%)	119 (64%)	86 (66%)	0.09
Diabetes	30 (16%)	34 (18%)	17 (13%)	0.46
Stroke	6 (3%)	2 (1%)	1 (0.8%)	0.08
Higher income	62 (36%)	80 (46%)	65 (51%)	0.006
Higher occupational complexity	94 (51%)	84 (44%)	84 (64%)	0.06
Higher education	108 (59%)	110 (59%)	84 (64%)	0.44

A Cochrane-Armitage test was performed for categorical variables, and general linear models were utilized for continuous variables. Higher income: annual household income > 33,333 USD. Higher occupational complexity: occupation with higher mental demands. Higher education: education > 12 years. SD = standard deviation; BMI = body mass index; CES-D = Center for Epidemiologic Studies Depression Scale; *APOE* = apolipoprotein E.

The independent effects of the 5 lifestyle factors and 3 SES indicators on cognitive change were further examined by adjusting for all of them simultaneously and other covariates. Increased fish intake protected against decline in global cognition and attention [β = 0.07, 95% confidence interval (CI) = 0.005–0.14; β = 0.12, 95% CI = 0.02–0.22, respectively, Table 4]. After controlling for lifestyles, higher income remained protective against decline in global cognition and logical memory (β = 0.14, 95% CI = 0.08–0.20; β = 0.32, 95% CI = 0.21–0.43, respectively), whereas higher occupational complexity protected against the decline of logical memory (β = 0.14, 95% CI = 0.03–0.26, Table 4).

**Table 4:** Adjusted effects of individual lifestyle factors and SES indicators on cognitive change.

	Cognitive variables				
Lifestyle factors N (%)	Global cognition	Logical memory	Executive function	Verbal fluency	Attention
	ß (95% CI)	ß (95% CI)	ß (95% CI)	ß (95% CI)	ß (95% CI)
Vegetables 152 (30%)	-0.0001 (-0.07, 0.07)	0.10 (-0.01, 0.22)	-0.10 (-0.21, 0.02)	-0.01 (-0.12, 0.09)	-0.01 (-0.11, 0.10)
Fish 143 (28%)	0.07 (0.005, 0.14)*	0.09 (-0.03, 0.21)	0.05 (-0.06, 0.17)	0.02 (-0.09, 0.13)	0.12 (0.02, 0.22)*
Physical act. 169 (33%)	0.009 (-0.06, 0.07)	0.03 (-0.08, 0.15)	0.05 (-0.06, 0.16)	0.02 (-0.09, 0.12)	0.01 (-0.09, 0.11)
Not smoking 494 (97%)	0.13 (-0.03, 0.30)	0.20 (-0.09, 0.50)	0.20 (-0.09, 0.49)	-0.11 (-0.37, 0.16)	0.17 (-0.08, 0.43)
Light to moderate alcohol 33 (6%)	0.09 (-0.03, 0.21)	0.18 (-0.04, 0.39)	0.20 (-0.01, 0.41)	0.10 (-0.10, 0.29)	-0.10 (-0.29, 0.09)
Higher income 208 (43%)	0.14 (0.08, 0.20)***	0.32 (0.21, 0.43)***	-0.03 (-0.13, 0.08)	0.008 (-0.09, 0.11)	0.02 (-0.07, 0.12)
Higher occupational complexity 264 (52%)	0.02 (-0.04, 0.08)	0.14 (0.03, 0.26)*	-0.10 (-0.21, 0.02)	-0.02 (-0.13, 0.08)	0.03 (-0.07, 0.13)
Higher education^a^ 304 (60%)	-0.002 (-0.07, 0.07)	0.06 (-0.07, 0.18)	0.04 (-0.08, 0.16)	0.02 (-0.09, 0.13)	0.10 (-0.01, 0.21)

The multivariable model was adjusted for age, sex, number of years of education, *APOE* ε4 status, baseline cognitive domain score, CES-D score, hypertension, diabetes mellitus, stroke, daily energy intake, occupational complexity, annual household income, and the five lifestyle factors.

^a^ Adjusted for the above covariates except substituting education level (> 12 years, ≤ 12 years) for number of years of education.

Higher income: annual household income > 33,333 USD. Higher occupational complexity: occupation with higher mental demands. Higher education: education > 12 years. CI = confidence interval; act = activity.

*P < 0.05,

***P < 0.001.

**Fig 1:**
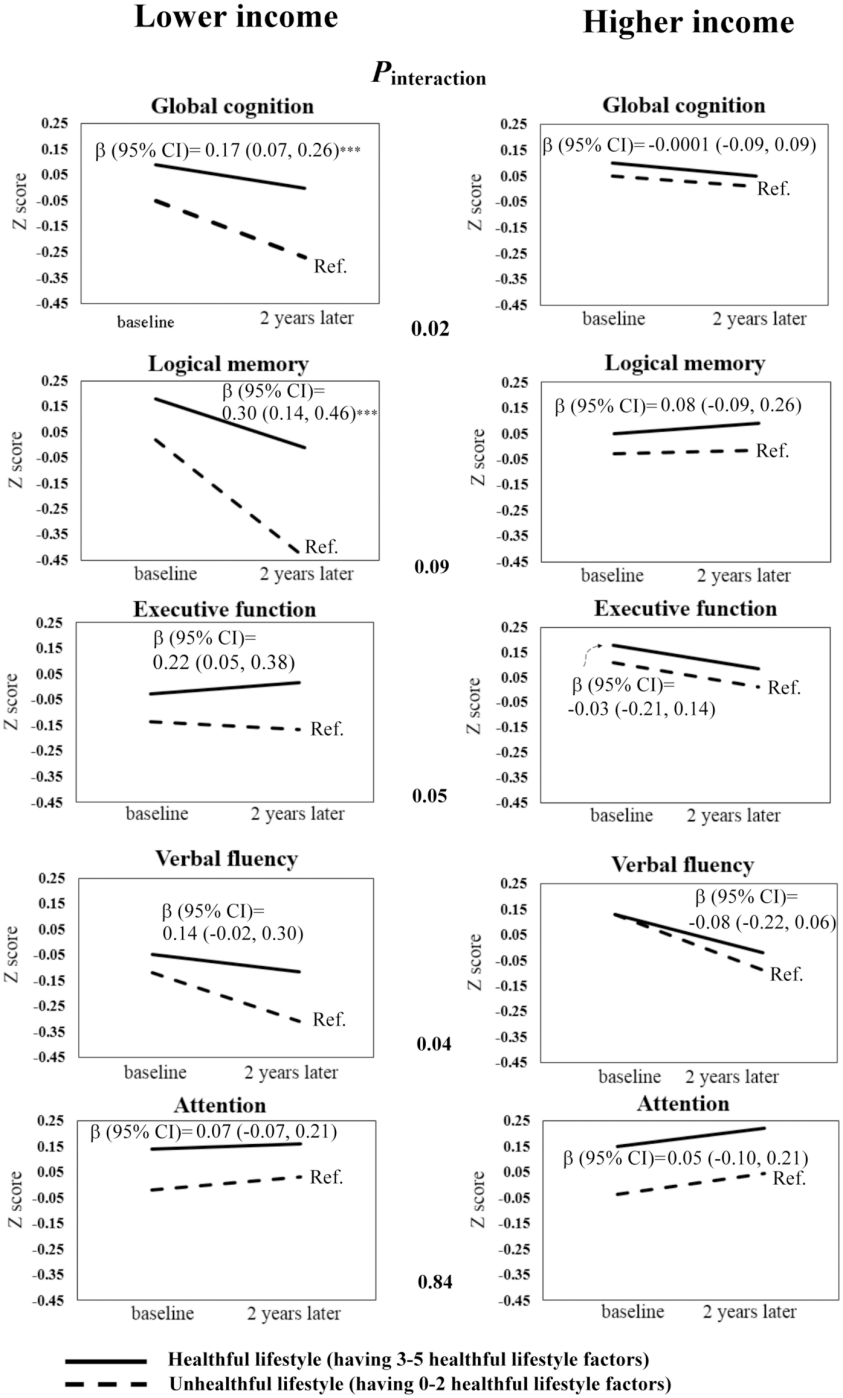
Interactions between a healthful lifestyle and annual household income on cognitive change. *P*_*interaction*_ is presented in the middle of the figure. After stratification by annual household income, the effects of having a healthful lifestyle compared with an unhealthful lifestyle are shown as β-coefficients (95% CI) above the solid lines after adjustment for age, sex, number of years of education, *APOE* ε4 status, baseline cognitive domain score, CES-D score, hypertension, diabetes mellitus, stroke, daily energy intake, and occupational complexity. The solid lines represent the cognitive change values for participants with a healthful lifestyle (having ≥ 3 healthful lifestyle factors), whereas the dashed lines represent individuals with an unhealthful lifestyle (having 0–2 healthful lifestyle factors). CI = confidence interval; Ref. = reference group. ***P < 0.001.

To examine the modifying effect of SES, the effects of a healthful lifestyle on cognitive change were examined by different SES categories (Fig 1). A healthful lifestyle was defined as having more than half of healthful lifestyle factors (≥ 3 out of 5). The crude cognitive change over 2 years was shown in Fig 1 after stratification by annual household income and lifestyles. Elders with lower income had greater cognitive decline (as shown by the steeper slopes) compared with those with high income. Among those with lower income, the cognitive decline was attenuated if they had a healthful lifestyle (as shown by the difference of slopes between the solid line and the dashed line, which represented the cognitive change for people with a healthful and unhealthful lifestyle, respectively). A healthful lifestyle was protective against the decline of global cognition (β = 0.17, 95% CI = 0.07–0.26) and logical memory (β = 0.30, 95% CI = 0.14–0.46) among elders with lower income but not those with a higher income. There was a significant interaction between a healthful lifestyle and annual household income on changes in global cognition and verbal fluency test (*P*_interaction_ = 0.02 and 0.04, respectively, Fig 1). No significant interactions were observed between a healthful lifestyle and occupational complexity or education level (S2 and S3 Figs).

## Discussion

To the best of our knowledge, the interaction between lifestyle and SES on cognitive change has not been explored previously. Five lifestyle factors and three SES indicators were shown to be protective against cognitive decline, especially fish consumption, higher income and higher occupational complexity. A significant interaction was observed between a healthful lifestyle and annual household income on cognitive change. Elders who were financially disadvantaged had the most cognitive benefit from a healthful lifestyle. This result may aid in the development of tailored lifestyle programs to prevent cognitive impairment and offer insights for policy making to decrease health inequalities caused by SES.

Several lifestyle factors were proved to be beneficial for cognition in this Chinese elderly population. We found that vegetables protected against cognitive decline but no effect was found for fruits, consistent with the findings of a previous systematic review [4]. One possible explanation is that vegetables contain more vitamin E than fruits, which was associated with decreased risk of Alzheimer’s disease [40]. Notably, we found that fish intake was the only significantly protective lifestyle factor after adjusting for the others. The omega-3 fatty acid in fish is essential for optimizing neuronal structure and function, and many studies found that the consumption of fish more than once per week protects against dementia [5]. The amount of fish intake is much higher in Taiwan (mean fish consumption: 3.8 servings/week in this study population) compared with that in most western countries, which might explain this more pronounced effect of fish. However, there are also concerns regarding the potential harms to the nervous and cardiovascular system from mercury and other heavy metals in certain types of fish [41]. More research should be undertaken before promoting fish intake above the current suggestion by the American Heart Association (2 servings/week) [42]. We also observed a borderline protective effect of physical activity in logical memory. The cognitive benefits of exercise have been well demonstrated previously [6, 7]. Because our study participants were recruited from elderly health checkup programs and, on average, had higher physical activity than their age-matched peers [43], the difference in cognitive change due to varied levels of physical activity may need follow-up longer than 2 years to be observable. We found that absence of current smoking is protective against cognitive decline, which is also in line with a meta-analysis [8]. In our study, former smoking did not have significant effect on cognitive change compared with never-smoking, probably because most of our former smokers quitted smoking many years ago (mean numbers of years since quitting: 23.6 years). Our findings were also consistent with most of previous studies that reported a U-shaped relationship between alcohol consumption and cognition, with light to moderate alcohol consumption being associated with decreased dementia risk compared with abstinence from alcohol or heavy drinking [11]. The effect of heavy drinking could not be examined because of a low proportion of heavy drinking (3.7%) in our population.

We also found that higher SES significantly and independently protected against cognitive decline. Several studies found that in addition to conventional biological or behavioral risk factors, SES is an important determinant of cognitive change [13–17]. Previous studies suggested that the effects of SES on health may be partly related to different lifestyles. Compared with high-SES populations, socioeconomically disadvantaged individuals tend to have poorer lifestyles, such as unable to afford high-quality food, inadequate intake of fruits, vegetables, and fish [19, 20], more cigarette smoking habits and inadequate exercise [20, 44]. However, SES remained significant even after adjusting for lifestyle factors, which may be attributable to the following reasons. First, higher SES is a proxy for higher cognitive reserve and may compensate for the neuropathological changes related to cognitive aging [21]. Second, material deprivation may play a role in the causal pathway of socioeconomic inequality and cognitive change. It is more difficult for socio-economically disadvantaged people to afford a good living environment, which is an important source of cognitive stimuli [45]. They may also have less access to good health services, leading to inadequate control of comorbidities. Last, people with lower SES tend to be more depressed [46]. The psychological stress resulting from perceptions of economic strain and social comparisons leads to faster aging in multiple physiological systems [47, 48].

In addition, a significant interaction was observed between lifestyle and income on cognitive change. The cognitive benefit of a healthful lifestyle was observed only among participants with lower income. Several studies reported that SES may act as an effect modifier between lifestyle and other health outcomes, including mortality and self-reported health [23, 49]. However, few studies examined how the interaction between lifestyle and SES affect cognitive change. Our findings are in line with Parrott’s study in Canada [50].They found that a prudent dietary pattern, characterized by vegetables, fruits, fish, poultry, and low-fat dairy products, was protective against cognitive decline only in elderly with low SES [50]. One possible explanation is that the adverse impact of an unhealthful lifestyle can be offset by high SES, which is a proxy for better cognitive reserve and may delay the manifestation of neuropathological changes [21]. On the contrary, the cumulative disadvantages for low-SES individuals, such as higher psychological stress and limited medical resources, makes them more vulnerable to the harm of bad lifestyles. Parrott’s study focused on the effects of dietary patterns, while our study further extended to more comprehensive lifestyle factors, e.g., physical activities, smoking, and alcohol use, etc. [50]. We found a significant interaction between lifestyle and income, but lifestyle did not significantly interact with occupational complexity or education. At different stage of life, SES levels may contribute differently in shaping cognitive reserve and preventing cognitive decline [13, 21]. Among the 3 indicators, income is the most proximal measure and may reflect the current and accumulated socio-economic position of the individual. Previous studies found that education-related disparities in health are attenuated in old age, whereas the health benefits associated with higher income persist throughout older age [51]. These may explain our findings. In addition, the educational level in this population was higher compared with the average Chinese elderly (mean years of education: 13.6 years), which may attenuate the difference related to education.

The findings of this study have several public health implications. The identified lifestyle factors may be useful for developing multidisciplinary dementia prevention programs given that different lifestyles may exert protective effect in a graded manner. Furthermore, we found that the effect of a healthful lifestyle was especially pronounced among economically disadvantaged individuals. People who were less financially privileged tended to have an unhealthful lifestyle, and they were particularly vulnerable to the adverse cognitive impacts by bad health behaviors. Thus, lifestyle programs targeting this disadvantaged group could be more cost-effective and may help to reduce health inequalities. For example, policies aimed at improving the diet quality in this high-risk group could be considered.

This study has several strengths. We screened an extensive list of lifestyle factors previously reported to be associated with cognitive decline and then identified protective lifestyle variables for Chinese elderly. A battery of validated neuropsychological tests were used to capture cognitive changes over time. We also attempted to minimize confounding effects by adjusting for other possible risk factors of cognitive decline, including *APOE* ε4 status, vascular comorbidities ascertained by self-reports, laboratory tests and medication use, along with SES indicators, which were often ignored in many lifestyle or social science studies [7, 8, 14].

This study has some limitations. Repeated neuropsychological tests may result in a learning effect, which may explain the improvement in the trail-making A test and digit span forward test after 2-year follow-up [52, 53]. Neuropsychological tests may also suffer from ceiling effect because the educational level in our population was higher than average, but we tried to increase discriminatory power by using 11 cognitive tests to assess performance in different cognitive domains. Additionally, 16% of elders were lost to follow-up. They were less educated and had a lower baseline cognitive score, which may lead to an underestimation of the lifestyle effect. This study examined the effect of late-life lifestyle, but the effect of a healthful lifestyle is on a long term basis which may occur decades before the onset of cognitive impairment. The pre-existing chronic diseases or functional status could also modify late-life lifestyle behaviors. However, the inclusion of ambulatory community-dwelling elderly without severe health problems may minimize this concern. Last, different food may have synergic effects or interaction on cognition, and dietary pattern would be a good alternative method to assess the dietary effect.

## Conclusions

In summary, we found that a healthful lifestyle based on a diet rich in vegetables and fish, increased physical activity, not smoking, and light to moderate alcohol consumption was beneficial to cognition in this Chinese elderly population. The effect of fish consumption was especially pronounced. Higher annual household income and occupational complexity were also independently associated with less cognitive decline. The protective effect of a healthful lifestyle was observed only among economically disadvantaged participants. Future large studies are needed to externally validate these findings. More attention needs to be given to analyze the effects of lifestyle on health under different socioeconomic circumstances and explore the underlying mechanisms.

## Supporting information

S1 Fig Association between number of healthful lifestyle factors and cognitive change. The graded relationship between the number of healthful lifestyle factors and cognitive change in different domains was examined with a multivariable linear regression adjusted for age, sex, number of years of education, *APOE* ε4 status, baseline cognitive domain score, CES-D score, hypertension, diabetes mellitus, stroke, daily energy intake, occupation, and annual income. Numbers in bold indicate significant findings. No., number; CI, confidence interval. *P < 0.05, **P < 0.01, ***P < 0.001.
(TIF)

S2 Fig Effects of interactions between healthful lifestyle and occupational complexity on cognitive change. *P*_*interaction*_ is presented in the middle of the figure. After stratification by occupational complexity, the effects of having a healthful lifestyle compared with an unhealthful lifestyle are shown as β-coefficients (95% confidence intervals) above the solid lines after adjustment for age, sex, number of years of education, *APOE* ε4 status, baseline cognitive domain score, CES-D score, hypertension, diabetes mellitus, stroke, daily energy intake, and occupation. The solid lines represent cognitive change values for participants with a healthful lifestyle (having 3–5 healthful lifestyle factors), whereas the dashed lines represent those for individuals with an unhealthful lifestyle (having 0–2 healthful lifestyle factors).CI = confidence interval; Ref. = reference group. *P < 0.05.
(TIF)

S3 Fig Effects of interactions between healthful lifestyle and education attainment on cognitive change. *P*_*interaction*_ is presented in the middle of the figure. After stratification by education attainment, the effects of having a healthful lifestyle compared with an unhealthful lifestyle are shown as β-coefficients (95% confidence intervals) above the solid lines after adjustment for age, sex, number of years of education, *APOE* ε4 status, baseline cognitive domain score, CES-D score, hypertension, diabetes mellitus, stroke, daily energy intake, and occupation. The solid lines represent cognitive change values for participants with a healthful lifestyle (having 3–5 healthful lifestyle factors), whereas the dashed lines represent those for individuals with an unhealthful lifestyle (having 0–2 healthful lifestyle factors). Higher education indicates education > 12 years. CI = confidence interval; HL-score = healthful lifestyle score; Ref. = reference group. *P < 0.05, **P < 0.01.
(TIF)

S1 Table Baseline characteristics by categories of socioeconomic status. (DOC)
