# Supplementary figures and images for: The effect of lifestyle on late-life cognitive change under different socioeconomic status
Source: PLoS One. 2018 Jun 13;13(6):e0197676. doi: 10.1371/journal.pone.0197676 (PMC5999076; doi:10.1371/journal.pone.0197676)

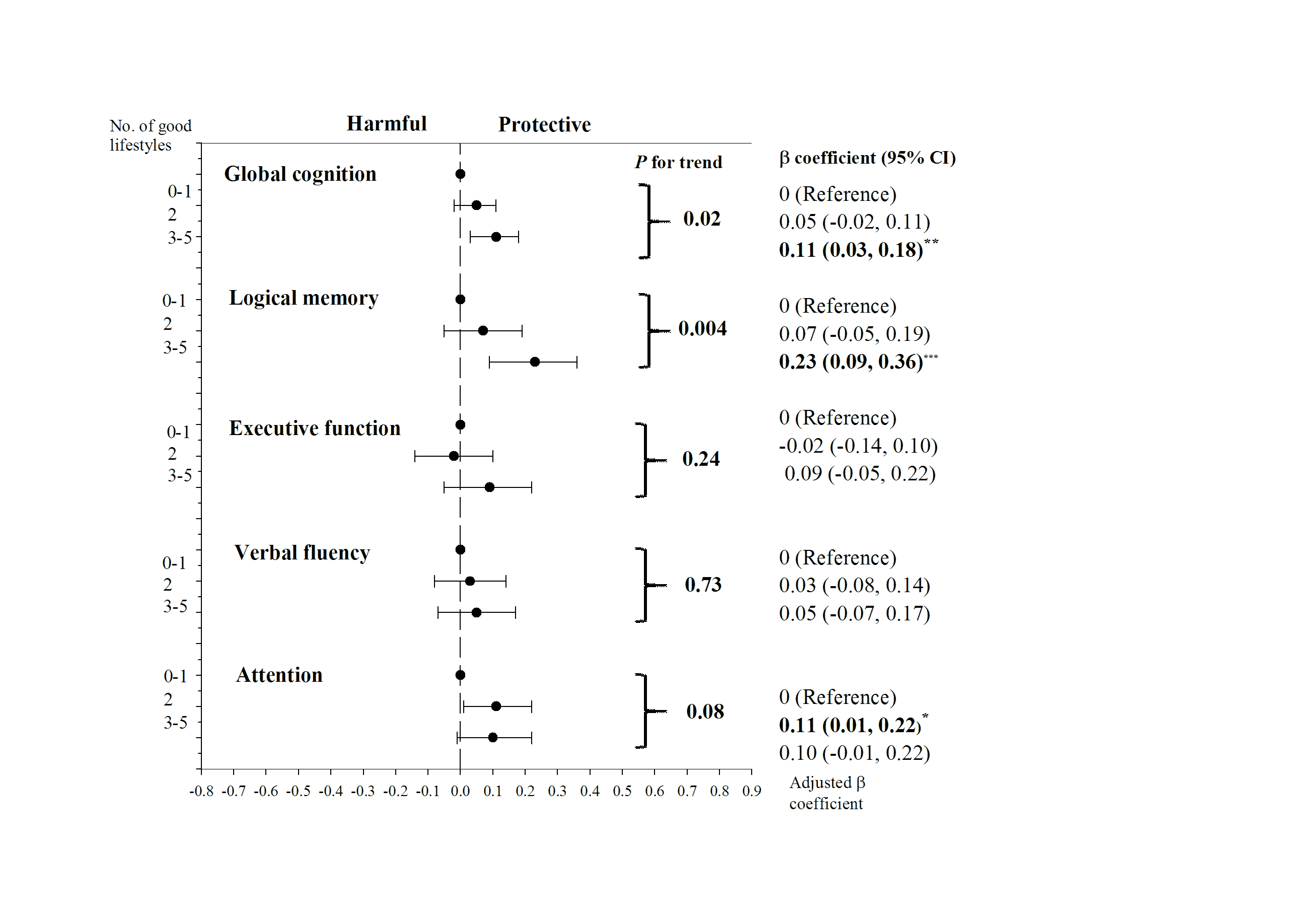

Supplement: S1 Fig — The graded relationship between the number of healthful lifestyle factors and cognitive change in different domains was examined with a multivariable linear regression adjusted for age, sex, number of years of education, APOE ε4 status, baseline cognitive domain score, CES-D score, hypertension, diabetes mellitus, stroke, daily energy intake, occupation, and annual income. Numbers in bold indicate significant findings. No., number; CI, confidence interval. *P < 0.05, **P < 0.01, ***P < 0.001. (TIF) [file pone.0197676.s001.tif]

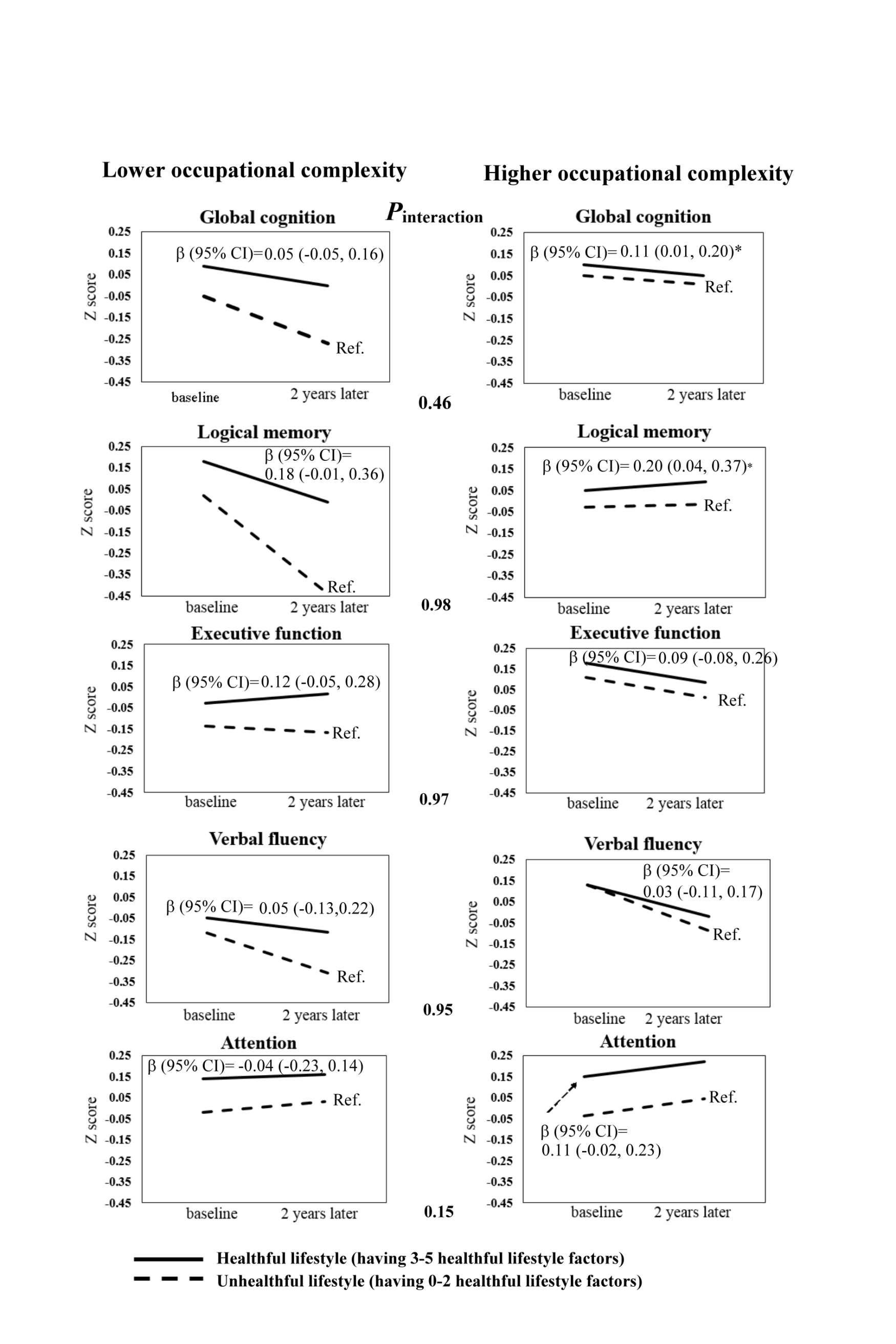

Supplement: S2 Fig — Pinteraction is presented in the middle of the figure. After stratification by occupational complexity, the effects of having a healthful lifestyle compared with an unhealthful lifestyle are shown as β-coefficients (95% confidence intervals) above the solid lines after adjustment for age, sex, number of years of education, APOE ε4 status, baseline cognitive domain score, CES-D score, hypertension, diabetes mellitus, stroke, daily energy intake, and occupation. The solid lines represent cognitive change values for participants with a healthful lifestyle (having 3–5 healthful lifestyle factors), whereas the dashed lines represent those for individuals with an unhealthful lifestyle (having 0–2 healthful lifestyle factors).CI = confidence interval; Ref. = reference group. *P < 0.05. (TIF) [file pone.0197676.s002.tif]

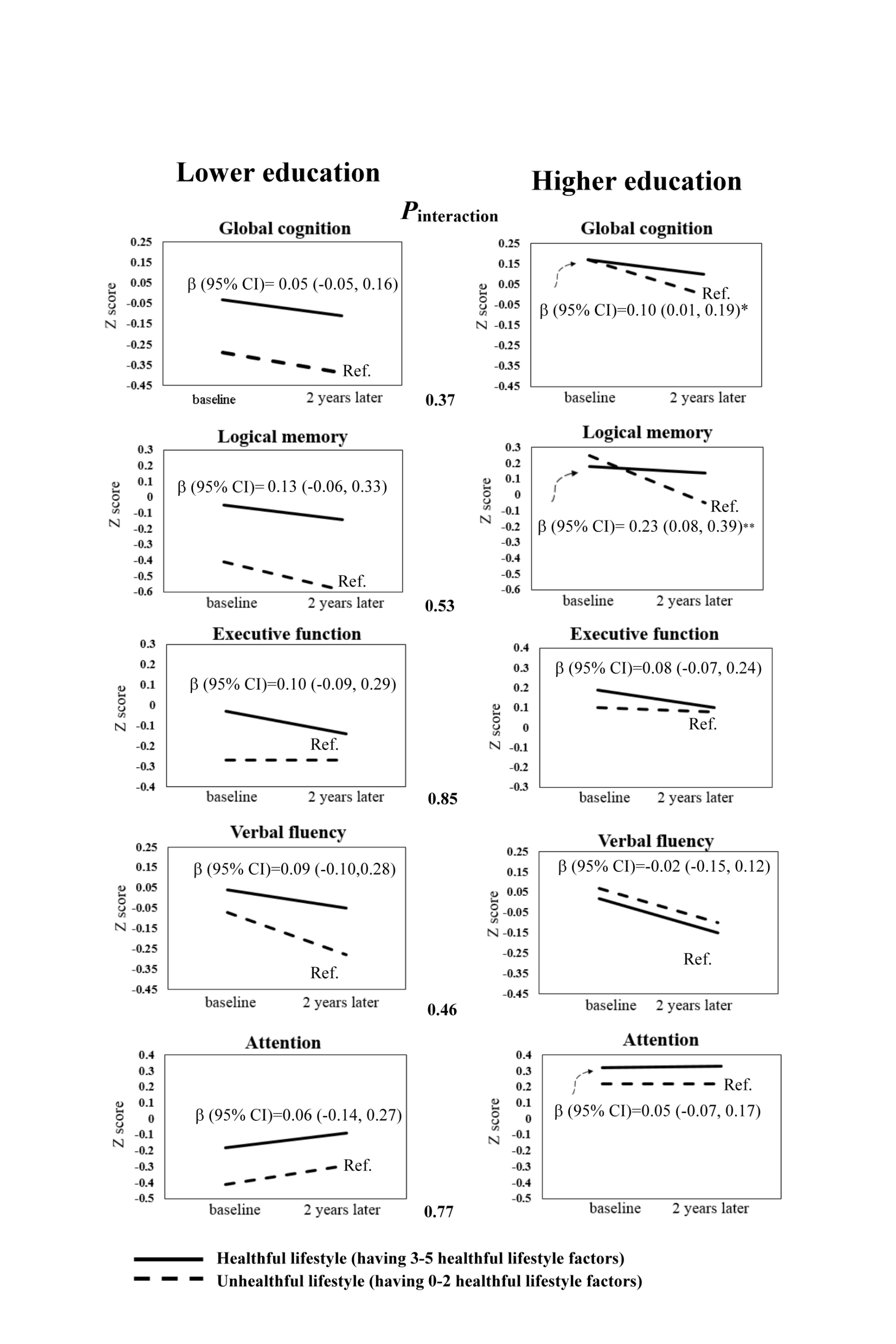

Supplement: S3 Fig — Pinteraction is presented in the middle of the figure. After stratification by education attainment, the effects of having a healthful lifestyle compared with an unhealthful lifestyle are shown as β-coefficients (95% confidence intervals) above the solid lines after adjustment for age, sex, number of years of education, APOE ε4 status, baseline cognitive domain score, CES-D score, hypertension, diabetes mellitus, stroke, daily energy intake, and occupation. The solid lines represent cognitive change values for participants with a healthful lifestyle (having 3–5 healthful lifestyle factors), whereas the dashed lines represent those for individuals with an unhealthful lifestyle (having 0–2 healthful lifestyle factors). Higher education indicates education > 12 years. CI = confidence interval; HL-score = healthful lifestyle score; Ref. = reference group. *P < 0.05, **P < 0.01. (TIF) [file pone.0197676.s003.tif]
