# Supplementary material for: The effect of lifestyle on late-life cognitive change under different socioeconomic status
Source: PLoS One. 2018 Jun 13;13(6):e0197676. doi: 10.1371/journal.pone.0197676 (PMC5999076; doi:10.1371/journal.pone.0197676)
Supplement: S1 Table — (DOC) [file pone.0197676.s004.doc]

**S1 Table. Baseline characteristics by categories of socioeconomic status.**

|  | **Socioeconomic status** | | | | | | | | |
| --- | --- | --- | --- | --- | --- | --- | --- | --- | --- |
|  | **Income** | | | **Occupational complexity** | | | | **Education** | |
|  | **Lower**  **(N=271)** | **Higher**  **(N=208)** | | **Lower**  **(N=245)** | | **Higher**  **(N=264)** | | **Lower**  **(N=204)** | **Higher**  **(N=304)** |
|  | **Mean ± SD** | | | | | | | | |
| Age (years) | 73.2 ±5.6 | 72.6 ± 5.3 | | 72.7 ± 5.3 | | 73.3 ± 5.5 | | 73.4 ± 5.5 | 72.7 ± 5.4 |
| BMI (kg/m^2^) | 24.1 ±3.1 | 23.8 ± 3.0 | | 23.9 ± 2.9 | | 24.0 ± 3.1 | | 24.2 ± 3.2 | 23.8 ± 2.9 |
| CES-D score | 3.4 ± 6.6 | 1.8 ± 4.8^***^ | | 3.1 ± 6.6 | | 2.5 ± 5.2 | | 3.5 ± 7.2^*^ | 2.4 ± 4.9^*^ |
|  | **N (%)** | | | | | | | | |
| Female sex | 142 (52%) | | 105 (50%) | | 164 (67%) | | 104 (39%)^***^ | 138 (68%) | 129 (42%)^***^ |
| *APOE ε*4 carriers | 41 (15%) | | 38 (19%) | | 41 (17%) | | 43 (17%) | 33 (16%) | 51 (17%) |
| Hypertension | 183 (69%) | | 135 (65%) | | 167 (70%) | | 172 (66%) | 135 (68%) | 203 (67%) |
| Diabetes | 39 (14%) | | 34 (16%) | | 41 (17%) | | 40 (15%) | 39 (19%) | 42 (14%) |
| Stroke | 4 (1%) | | 5 (2%) | | 5 (2%) | | 4 (2%) | 6 (3%) | 3 (1%) |
| Vegetables | 57(21%) | | 91(44%)^***^ | | 63 (26%) | | 89 (34%) | 56 (28%) | 95 (31%) |
| Fish | 85 (32%) | | 50 (24%) | | 65 (27%) | | 78 (30%) | 55 (27%) | 88 (29%) |
| Physical activity | 84 (31%) | | 72 (35%) | | 79 (32%) | | 90 (34%) | 66 (32%) | 103 (34%) |
| Not smoking | 85 (96%) | | 203 (98%) | | 238 (97%) | | 256 (97%) | 201 (99%) | 292 (96%) |
| Light to moderate alcohol | 14 (5%) | | 19 (9%) | | 15 (6%) | | 18 (7%) | 8 (4%) | 25 (8%) |
| Higher income | NA | | NA | | 85 (38%) | | 123 (48%) | 70 (38%) | 138 (47%) |
| Higher occupational complexity | 135 (50%) | | 123 (59%) | | NA | | NA | 54 (26%) | 210 (69%)^***^ |
| Higher education | 157 (58%) | | 138 (66%) | | 94 (39%) | | 210 (80%)^***^ | NA | NA |

Chi-square tests were performed for categorical variables. For continuous variables, Student’s T-tests or Mann-Whitney U tests were performed for data with or without normal distributions, respectively. Higher income: annual household income > 33,333 USD. Higher occupational complexity: occupation with higher mental demands. Higher education: education > 12 years. SD = standard deviation; BMI = body mass index; CES-D = Center for Epidemiologic Studies Depression Scale; *APOE* = apolipoprotein E; NA = not applicable. ^*^*P* < 0.05, ^**^*P* < 0.01, ^***^*P* < 0.001.
